# Effectiveness of music therapy for alleviating pain during haemodialysis access cannulation for patients undergoing haemodialysis: a multi-facility, single-blind, randomised controlled trial

**DOI:** 10.1186/s13063-019-3773-x

**Published:** 2019-11-19

**Authors:** Masatsugu Kishida, Yosuke Yamada, Emi Inayama, Mineaki Kitamura, Tomoya Nishino, Keiko Ota, Ayumi Shintani, Tatsuyoshi Ikenoue

**Affiliations:** 1 Division of Nephrology, National Cerebral and Cardiovascular Center, 6-1 Kishibeshinmachi, Suita, 564-8565 Japan; 2 Department of Nephrology, Shinshu University School of Medicine, 3-1-1 Asahi, Matsumoto, 390-8621 Japan; 3 Mihama Narita Clinic, 129-1 Iida-cho, Narita, 286-0041 Japan; 4 Department of Nephrology, Nagasaki University Hospital, 1-7-1 Sakamoto, Nagasaki, 852-8501 Japan; 5 Department of Medical Statistics, Osaka City University Graduate School of Medicine, 1-4-3 Asahi-cho, Osaka, 545-0051 Japan; 6 Graduate School of Medicine and Public Health, Kyoto University, 53 Kawahara-cho, Sakyo-ku, Kyoto, 606-8507 Japan

**Keywords:** Music therapy, Cannulation, Pain, Anxiety, Visual Analogue Scale, Haemodialysis

## Abstract

**Background:**

Repeated pain during haemodialysis access cannulations is a serious problem for haemodialysis patients even when prescribed oral or topical analgesics. Although some studies have observed the efficacy of music therapy for improving pain and anxiety, its effectiveness during haemodialysis access cannulations during dialysis is uncertain. The purpose of this study is to investigate the effects of music therapy for pain when cannulating haemodialysis access for haemodialysis patients.

**Methods:**

A prospective, multi-facility, single-blind, crossover, randomised controlled trial will be implemented. The intervention includes listening to Mozart, along with a white noise control condition. One hundred twenty haemodialysis patients will be enrolled across five facilities. Patients will be randomly allocated to either an Early-sequence group or a Later-sequence group. The Early-sequence group will receive cannulation while listening to Mozart’s Sonata for two pianos in D major (K.448) during the second week (Music period) and white noise during the fourth week (White noise period). The Later-sequence group will receive cannulation along with white noise first, followed by Mozart. All patients will also undergo cannulation during a no-sound period (wearing only headphones) during the first and third week (No-sound period). The music or no-music protocol will begin 8 min prior to the cannulating procedure, and participants will finish listening after starting haemodialysis during each period. The primary outcomes that will be assessed include the Visual Analogue Scale (VAS) score for pain during cannulation, and secondary outcomes are blood pressure, heart rate, VAS anxiety score, State-Trait Anxiety Inventory score, and salivary amylase activity. The operators who are in charge of haemodialysis access cannulation will be blind to the listening condition and VAS report.

**Discussion:**

The proposed study has several methodological benefits. First, using white noise is a suitable control condition for addressing the role of sound in pain management. Additionally, using a crossover design with repeated measurements can help control individual differences between participants, which should better distinguish between- and within-participant variability. Overall, music therapy is a safe and inexpensive intervention that does not have the problematic side effects typically associated with pharmacological treatment. If effective, music therapy can be easily implemented for reducing pain and anxiety during cannulation.

**Trial registration:**

This trial was prospectively registered to UMIN Clinical Trials Registry on 1 July 2018 (UMIN 000032850).

## Background

When kidney disease reaches its end stage, most patients undergo haemodialysis at least three times per week for life support. At the beginning of each session, two cannulations are required to be made. One is for removing blood from patients’ haemodialysis access, and the other is for returning blood to it. As such, patients often experience a significant amount of pain. This pain can be particularly problematic, especially given that many haemodialysis patients often undergo this procedure at least 150 times in a year [1–3]. Although several medications are available to alleviate this pain, 19.5% of haemodialysis patients experience severe pain [4]. Oral or topical analgesics are the most common treatment modality [5–7]. However, these medications can produce unwanted side effects (such as dermatitis); thus, they are not appropriate for all patients [8–12]. Moreover, aspects of cannulation pain cannot be easily reduced via oral or topical analgesics (i.e., feelings of anxiety or depression or both) [13–16]. However, certain sedatives or antidepressants, which may improve symptoms of anxiety or depression or both, can increase one’s risk of hypotension and physical dependence [17–20]. In addition, prescribing analgesics, sedatives or antidepressants can be expensive [21] and such expenses can be a serious issue for patients and insurers [22–24].

Given the aforementioned limitations of pharmacological treatment, the present study advocates the use of music therapy for pain management. Music therapy is safe and inexpensive and has been shown to improve pain and anxiety associated with several diseases [25–29]. In terms of cancer-related pain within a perioperative setting, music therapy helps alleviate pain, as noted by significant self-reported reductions in pain via Visual Analogue Scales (VASs) [30]. Music therapy is also effective for reducing lumbar puncture pain [31], and among haemodialysis patients, music therapy helps alleviate headaches, chest pain, and back pain resulting from disequilibrium syndrome [32]. Finally, music therapy has been effective for relieving pain during blood vessel cannulation procedures, given that such pain often derives from anxiety regarding the procedure [33–36]. However, no prior study has examined the effectiveness of music therapy on pain during haemodialysis access cannulations for haemodialysis patients.

The proposed study is a crossover, randomised controlled trial investigating the effectiveness of music therapy on pain during haemodialysis access cannulations administered to haemodialysis patients. Many previous studies assessing the effectiveness of music used only a no-sound control group [37], which could produce a potential ‘placebo effect’ for the intervention. For instance, music therapy effect sizes range from 26% to 58%, and the average effect is 35% [38, 39]. Given the placebo effect, the proposed study will use white noise as the control in order to better extract the true effect of music therapy.

## Methods/Design

Our main purpose for this trial is to clarify whether music therapy can truly alleviate pain, measured using a VAS metric, during cannulation into a haemodialysis access point, as compared with white noise. This protocol has been reported according to the Standard Protocol Items: Recommendations for Interventional Trials (SPIRIT) guidelines 2013 (see Additional file 1 for details).

### Design (Fig. 1)

This study will be a prospective, multi-facility, single-blind, crossover, randomised controlled trial. Haemodialysis patients will be allocated randomly and equally to either an Early-sequence or Later-sequence group. For the Early-sequence group, patients will receive cannulation while not listening to sound (only wearing headphones) during the first (run-in) week (No-sound period), listen to music during the second week (music period), listen to no-sound during the third (washout) week (No-sound period), and listen to white noise during the fourth week (White noise period). For the Later-sequence group, the no-sound period commences in the first (run-in) week, white noise during the second week, no-sound during the third (washout) week, and music during the fourth week.

**Fig. 1:**
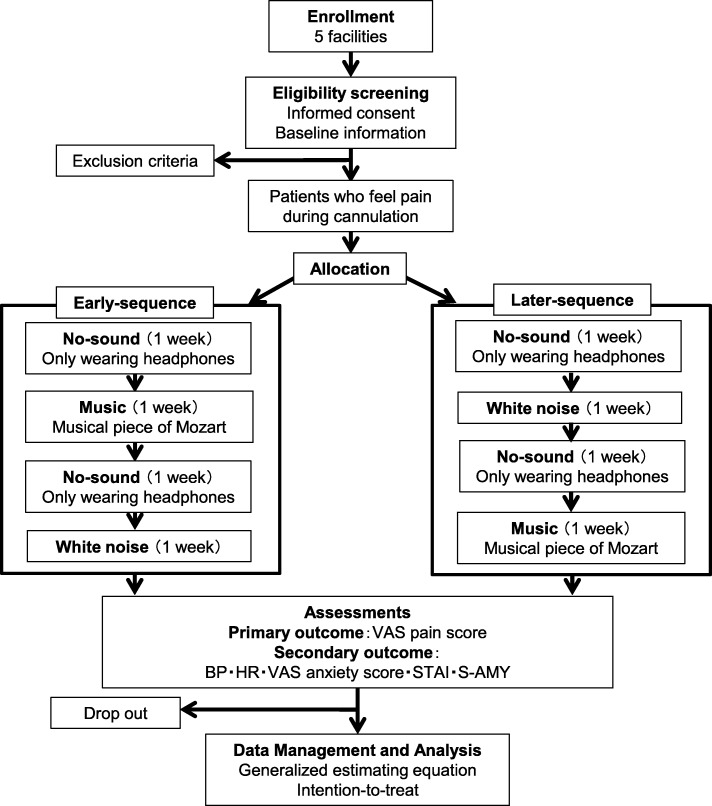
Trial workflow. There are four periods, one week each, for a total of four weeks

### Setting

This trial will be performed at five outpatient maintenance haemodialysis facilities: Jisyukai Ueda Kidney Clinic (Nagano, Japan), Nagasaki Renal Center (Nagasaki, Japan), Fujiidera Keijinkai Clinic (Osaka, Japan), Mihama Narita Clinic (Chiba, Japan), and Hakuyu Chiyoda Clinic (Osaka, Japan). The staff in all facilities will receive training on the trial method and protocol.

### Enrolment

Across the five facilities, patients over the age of 20 years who are undergoing outpatient haemodialysis three times a week, who have received dialysis for more than 6 months, and who indicated experiencing pain during cannulation based on a prior questionnaire will be enrolled. To evaluate the patients’ pain status, we will not adopt the VAS but rather a simple categorical scale questionnaire that will enable us to exclude patients who do not experience any pain (with a VAS of 0 mm) throughout the week of the intervention. The questionnaire is as follows:

Q-1) Do you feel pain when your haemodialysis access is cannulated?

Answer) A. Always; B. Sometimes; C. Never.

Q-2) Please answer Q-2 if you answered B on Q-1. About how often do you feel pain?

Answer) A. Once a week or more; B. Less than once a week.

Patients who answer A on Q-1, B on Q-1, and A on Q-2 are considered to have experienced pain and are eligible to participate. The use of analgesics is allowed throughout this trial.

#### Exclusion criteria

The exclusion criteria are as follows: not willing to participate; having a hearing, writing, or visual impairment; being paralysed; facing a difficulty communicating; having a psychiatric disorder or dementia; undergoing haemodialysis therapy fewer than three times per week; and receiving dialysis through an indwelling catheter.

### Trial interventions

**Fig. 2:**
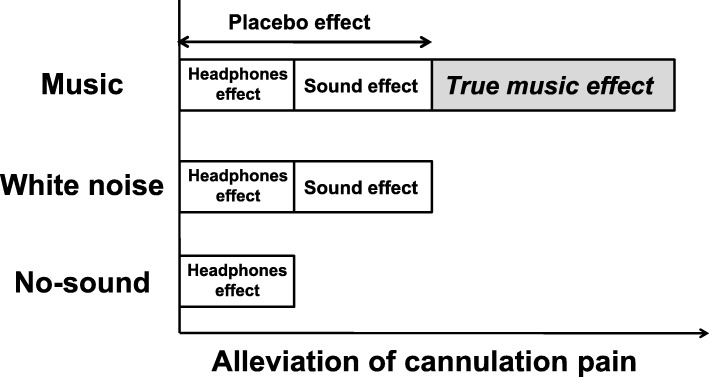
Schematic diagram of intervention efficacy. The analgesic effects of music therapy are thought to include not only the true effect of music therapy but also two placebo effects (Headphones effect and Sound effect). Thus, music therapy will be set as the intervention, and white noise will be set as the control, in order to extract the true pain-alleviating effect of music therapy

The intervention for this protocol is referred to as ‘listening to sound’. The protocol will be performed while the patient is on a bed. The patient wears headphones (JVC HA - S 88 BN; JVC Kenwood Co., Kanagawa, Japan) connected to a tablet PC (BNT - 791 W.; Bluedot Co., Chiba, Japan). The sound will be provided by the Research Electronic Data Capture (REDCap) system (version 8.1.13; Vanderbilt University, Nashville, TN, USA) on a tablet PC. When the intervention is started, the participant will adjust the volume on the tablet PC screen. To reduce noise other than the sound from the headphones, we will use a noise-cancelling function. The specific intervention is outlined below (Fig. 2).

#### Music

For this condition, patients will listen to music during cannulation. Mozart’s Sonata for two pianos in D major (K.448), which has been verified as the ‘Mozart effect’ in several studies [40–46], will be used. Eight minutes after listening to the piece, the operator will begin the puncture procedure (including disinfection, puncture, and blood removal). The patient will then finish listening to the piece after the punctures. There are two reasons for starting the music-listening period 8 min before the procedure. First, Sonata 2 major includes a first movement (‘Allegro con spirit D major 4/4 beat sonata form’), a second movement (‘Andante G major three quartz sonata form’), and a third movement (‘Molto allegro D major 2/4 of the beat Rondo Section’). At about the eighth minute, the song modulates from the first movement (with a fast tempo) to the second movement (with a slow tempo). Here, we expect that a relaxation effect could be expressed during the slow tempo of the second movement [37]. Second, in previous studies examining the Mozart effect, participants listen to the piece for 10 min [37]. During blood vessel cannulation for dialysis, two or more punctures are performed (removing and returning blood). Thus, in order to evaluate the most intense pain between punctures, it is difficult to set a strict timing. A previous study indicated that the puncture procedure takes about 4 min [47]. Therefore, we confirmed the actual puncture time needed in the pilot study. We observed that the puncture could be performed in about 10 min if the procedure began 8 min after the start of the piece; hence, the initial music period was set to 8 min. However, the timing can change when cannulation failure and other unforeseeable circumstances occur. There is a possibility that the effect of music therapy will vary depending on the listening time from music onset to cannulation. Therefore, we will record the time of the sound onset and the end of the intervention using REDCap. The occurrence of cannulation failure will also be recorded as conditional information regarding the cannulation. Hence, we will be able to perform sensitivity analyses using these data.

#### White noise

During the white-noise period, the patient will listen to white noise during cannulation [48]. White noise is defined as sound comprising the same intensity of all frequencies within the range of human audition (1–22.05 KHz) [30, 49]. (The sound can be heard in https://www.youtube.com/watch?v=_CMzWGteDCY.) White noise has no orderly arrangement regarding melody, harmony, rhythm, tone or pitch, which is required for a sound to be considered musical [50, 51]. White noise was chosen as a control condition to isolate the effect of wearing headphones (Headphones effect) and the effect of stimulating hearing with sound (Sound effect).

#### No-sound

The no-sound period includes attaching headphones with no sound present. Outer noise is still cancelled out by the headphones [52]. This intervention will be used during the run-in and washout periods. During this period, we will attempt to diminish the placebo effect by using headphones.

### Study protocol

Figure 1 outlines the trial protocol and randomisation procedure. The intervention will be performed during each study period from the time of cannulation to the start of dialysis (thrice a week) (Table 1).

**Table 1:** Summary of collected data at each time point according to SPIRIT 2013 guideline

TIME POINT	Enrolment HD1	1 week			1 week			1 week			1 week		
		HD2	HD3	HD4	HD5	HD6	HD7	HD8	HD9	HD10	HD11	HD12	HD13
ENROLMENT													
Eligibility screening	×												
Informed consent	×												
Allocation	×												
INTERVENTIONS													
Early-sequence													
No-sound		×	×	×				×	×	×			
Music					×	×	×						
White noise											×	×	×
Later-sequence													
No-sound		×	×	×				×	×	×			
Music											×	×	×
White noise					×	×	×						
ASSESSMENTS													
Baseline information	×												
VAS pain score		×	×	×	×	×	×	×	×	×	×	×	×
VAS anxiety score		×	×	×	×	×	×	×	×	×	×	×	×
STAI Y-1				×			×			×			×
STAI Y-2	×												
BP and HR		×	×	×	×	×	×	×	×	×	×	×	×
S-AMY				×			×			×			×
Conditional information		×	×	×	×	×	×	×	×	×	×	×	×
Adverse events		×	×	×	×	×	×	×	×	×	×	×	×

*Abbreviations*: *BP* blood pressure, *HD* haemodialysis, *HR* heart rate, *S-AMY* salivary amylase activity, *SPIRIT* Standard Protocol Items: Recommendations for Interventional Trials, *STAI* State-Trait Anxiety Inventory, *VAS* Visual Analogue Scale

**Fig. 3:**
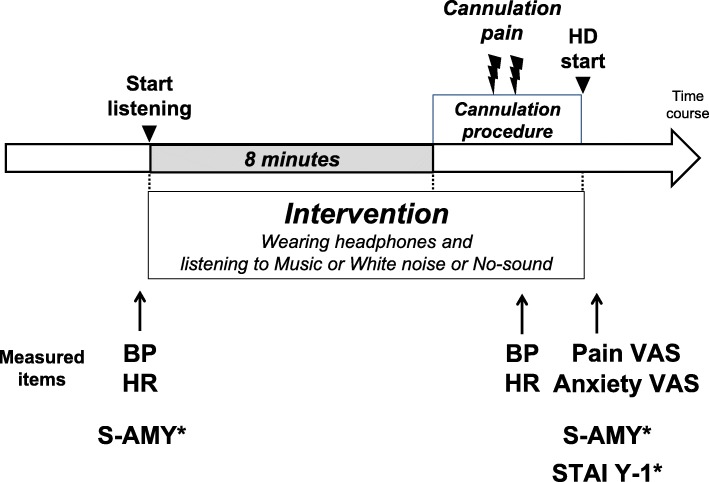
Data collection scheme. Each intervention will be performed for 8 min prior to starting the cannulation procedure to just after starting haemodialysis. *Will be measured only on HD4, HD7, HD10 and HD13

Figure 3 shows the flow of the interventions performed each day during implementation. First, blood pressure (BP), heart rate (HR) and salivary amylase activity (S-AMY) will be measured. Then, the patient wears the headphones connected to the tablet PC. Eight minutes after the initial listening period (Music, White noise periods or No-sound), the operator will prepare for the haemodialysis access cannulation and then cannulate the haemodialysis access. Immediately after the cannulation, BP and HR will be measured again. After connecting the cannula to the haemodialysis machine, the patient will stop listening and report his or her VAS pain score, VAS anxiety score, and State-Trait Anxiety Inventory (STAI). Both the time from the start of listening to the sound and the time of the end of the intervention will be recorded by REDCap. The patients enter his or her VAS pain score, VAS anxiety score, and STAI directly into the REDCap system on a tablet PC. This procedure ensures data independence, as the operator and investigator will be blinded to patients’ reports. S-AMY is also measured at this time; however, S-AMY and STAI are assessed only during haemodialysis (HD)4, HD7, HD10 and HD13.

### Data collection and follow-up

#### HD1

##### Baseline information

Baseline information—including age, gender, height, weight, duration of haemodialysis, history of smoking, medication, type and location of blood access, cause of kidney dysfunction, history of diseases (including skin disease, chronic heart failure, collagen disease, diabetic complications, liver disease, cancer, and AIDS, which are used to calculate Charlson Comorbidity Index [53]) and music preferences—will be collected.

##### State-Trait Anxiety Inventory (STAI) -Y-2

The STAI is a commonly used measure to assess trait and state anxiety [54–56]. The State-Trait Anxiety Inventory-JYZ (Jitsumukyoiku-Shuppan Co., Ltd., Tokyo, Japan) will be used. Each patient will complete this measure on a tablet PC. The STAI comprises 40 questions. The first 20 measure state anxiety, and the latter 20 measure trait anxiety. This measure has been previously validated for assessing anxiety during music therapy [57]. Whereas state anxiety can be considered more situational in nature and often is associated with the arousal of the autonomic nervous system, trait anxiety can be thought of as a worldview that an individual uses when coping with situations in his or her environment [58]. The first 20 items will be administered during each session, whereas the latter 20 will be administered only at HD1.

#### HD2–13

##### Conditional information regarding the cannulation

Conditional information regarding the cannulation will also be collected. This includes planned removal of water volume, weather (reported to have a relationship with the status of anxiety [59, 60]), use of topical analgesics, use of oral analgesics within 6 hours prior to the cannulation, the number of years that the operator has conducted dialysis, operator’s occupation (doctor, medical engineer, or nurse), gauges of the needle used for cannulation (on both the removing side and returning side), cannulation needle shape, number of cannulations (occurrence of cannulation failure), single needle used, change of operator, and posture when the patient was punctured.

##### Primary outcomes

The primary outcome is the VAS pain score during the cannulation. The VAS comprises a 100-mm line. The leftmost value is 0, which indicates no pain. The rightmost value is 100, which indicates maximum pain. Patients mark a point with their finger on the screen [61]. Although patients receive at least two cannulations during each dialysis session, pain will be evaluated only once per session. The maximum pain of the cannulations will be scored.

##### Secondary outcomes

The secondary outcomes include BP, HR, VAS anxiety score (evaluated on HD2–HD13), STAI (state scale: Y-1) and S-AMY (evaluated on HD4, HD7, HD10 and HD13).

BP and HR will be measured immediately after putting on the headphones and after the puncture procedure (once each time) via an electronic sphygmomanometer attached to the dialysis console. Each patient will be measured in the same posture (sitting up or supine) throughout the procedure.

Anxiety will be assessed via VAS on a tablet PC just after starting haemodialysis. The anxiety scale is in the same format as the pain scale and will be completed in the same manner [61].

Patients’ state anxiety via the STAI (Y-1) will be assessed immediately after the puncture during the No-sound period, Music period, and White noise period, respectively.

S-AMY fluctuates on the basis of autonomic nervous system activity, thus making it a reliable and objective marker of mental and physical stress [62–64]. With S-AMY, anxiety can be evaluated not only subjectively by VAS and STAI but also objectively. A salivary amylase monitor (Nipro, Co., Osaka, Japan) and a corresponding test strip (Nipro, Co.) will be used. Saliva is collected by a test strip placed under the tongue for about 30 s, and amylase concentration in the saliva is immediately measured.

S-AMY levels will be evaluated prior to and after the cannulation at the end of the No-sound period, Music period, and White noise period, respectively. After each intervention period, adverse events will be assessed.

### Safety assessments

As cannulations are a regular procedure in a haemodialysis session and listening to sounds is not a major invasive procedure, there is no expected harm for patients to participate in this intervention, as no negative effects of music therapy have been reported in prior studies. The saliva collection method is also harmless. Nevertheless, participant safety will be ensured during the protocol.

### Sample size

We conducted a pilot two-arm randomised controlled trial at four facilities, whereby eight haemodialysis patients were assigned randomly into either of the two groups: Group 1 listened to Mozart, and Group 2 listened to the news on the radio. The primary outcome was cannulation pain evaluated via a VAS. A previous review suggested that patients who record a baseline VAS score of more than 30 mm might indicate at least moderate pain [65]. Moura and colleagues reported that 20% of the haemodialysis patients suffer from pain, indicated by a VAS pain score of more than 30 mm [4]. In our pilot study, the mean VAS results were 20.5 mm in Group 1 and 25.4 mm in Group 2. All VAS scores were normally distributed, and the standard deviation was 12.0 mm. Based on results of clinical trials of analgesic agents [66, 67] and a cost-benefit performance of music therapy, a difference of 4.9 mm was considered to be clinically significant and was used for the sample size design. From these results, we computed that 95 patients are needed to observe a treatment effect based on this effect size at a power of 80% with a two-sided significance level of 5%. Assuming participant attrition, we will recruit a total of 120 participants. As the proposed study is a crossover trial wherein the statistical power is assumed to be greater than that of a two-arm design, we believe that our study is adequately powered.

### Randomisation

Haemodialysis patients who meet all inclusion criteria and do not meet any of the exclusion criteria will be randomly assigned to the Early-sequence group or the Later-sequence group during the enrolment period (HD1). The permuted block randomisation will be performed by using REDCap [53, 54]. Only selected staff at each facility will be authorised to access REDCap for patient randomisation.

### Blinding procedure

We will explain to the patients that the first period and third period are no-sound periods and that the second period and the fourth period are sound-listening periods (music or white noise) when we are obtaining informed consent. Operators will also receive these explanations in advance; moreover, during the no-sound period, they will be informed that it is a no-sound period on the tablet’s screen. The reason for such an explanation is to prevent patients from mistakenly believing it is a no-sound period if equipment malfunctions or if the volume of the sound is too low in the initial setting. Of course, patients and operators will not know in advance the results of allocation (i.e., arrangement of music period and white noise period in the second and the fourth week) to maintain study blindness.

Blinding participants to the allocation and evaluation of the VAS and STAI scores is not possible, as patients will be aware of what they will be hearing and the specific evaluations conducted. However, if participants are told that the purpose of this study is to assess the effectiveness of the music therapy, a demand characteristic, which refers to participants playing the role of a ‘good participant’ by altering their behaviour in order to obtain the researchers’ expected outcome, could emerge, resulting in information bias [68, 69]. Thus, we will not tell participants that we are assessing the effectiveness of a music intervention per se. In essence, participants will not be aware of whether music or white noise will be more soothing. Specifically, we will state, ‘Both sounds are considered effective, and we will examine which one is more effective’, in order to alleviate any potential demand characteristics.

As the patients will not be blinded to the treatment allocation, it is important that the person evaluating the outcome measure of VAS be blinded to the study. Although the outcome evaluator needs to also perform the randomisation, we created an automated process in REDCap to randomly choose and play either the music or the white noise without letting the study personnel know which sound is being played. Along with randomisation, they will be blinded to the music operation and evaluation procedures to measure the VAS as follows. The operator will dispense the sounds from REDCap; however, the REDCap screen will not indicate whether music or white noise is being played. Participants will wear a headset while the music or white noise is being played, and they will be instructed not to tell the study personnel what sound is being played. The patients will provide VAS and STAI scores via an online questionnaire via REDCap. Therefore, the operator will not see these results. Each haemodialysis facility will have designated staff with access to the REDCap and enter the data.

### Data management

A software toolset and workflow methodology for electronic data collection and management (REDCap) will be used. REDCap servers are managed by Osaka City University using cloud servers. All web-based information transmission will be encrypted. All protocols, consent forms, and data will be stored in the REDCap system. Data monitoring will be performed by an independent data coordinating centre. Two independent statisticians will be able to access the trial dataset.

### Analyses

#### Analysis of VAS pain scores during the cannulation

A set of outcome variables, which includes the VAS pain score as the primary outcome and the VAS anxiety score, STAI score, and S-AMY as the secondary outcomes, will be assessed during a single dialysis session. Each study period will last for a week including three dialyses. Patients will undergo two study periods; thus, each patient will be assessed for six sets of the outcome variables (three in the music period and other three in the white noise period).

A linear mixed model will be applied to compare the means of the three repeated outcome measures between the music and the white noise periods. For the outcome that accompanies the baseline measures, the mixed model will compare the mean of three repeated change scores between the two periods. Compound symmetry was used for variance-covariance to estimate dependency among the repeated measures. Two-sided significance level will be set at 0.05. The normality of the residuals will be confirmed via Q-Q plots, and the mathematical transformation of the outcome variable will be performed if necessary. The mixed-model approach was chosen on the basis of a Monte Carlo simulation study in order to compare the statistical power of three different analytical strategies, which include the following (see Additional file 2).

1. Paired *t* test to compare the two means of three pain scores between the two periods. (The unit of observation in the analysis is the within-patient mean score of the three scores; the analysis includes two mean scores per patient.)
2. Linear mixed model to compare the two means of the three pain scores between the groups. (The unit of observation is the same as 1.)
3. Linear mixed model to compare the six pain scores between the two periods. (The unit of observation is the pain scores; the analysis includes six observations per patient.)

The third approach was chosen because it appeared to be superior to other approaches, as it provides the largest statistical power to detect statistical significance while controlling for type I error. In addition, the direct inclusion of all six observations will allow us to control for time-dependent confounders, such as personnel effect from the operator who performs the cannulation.

#### Interim analyses

As mentioned above, participation in this study has no known harm for the patients. Interim analyses will not be performed.

#### Analysis set

A complete or full analysis set (FAS) will be used. FAS is obtained when participants are allocated to the Early-sequence or Later-sequence after eligibility, and VAS pain scores are available at one or more cannulations during the music or white noise conditions. Furthermore, a target group conforming to the implementation plan is defined as per protocol set (PPS). For PPS, when an observation is discontinued by stopping the protocol, the subsequent data are not used in the analysis. Analysis of primary and secondary outcomes is the main focus of FAS. We also will perform analyses targeting PPS so as to confirm the stability of the analytic outcomes.

### Monitoring

An independent data coordinating centre will monitor the input data by using the data log on REDCap, which records the input time and listening time.

### Consent withdrawal/dropout/missing data

#### Consent withdrawal

When a participant withdraws consent, we will discard all data gathered from the study initiation to the withdrawal date.

#### Dropout

Data collection for a participant will be discontinued, and data collected from the study initiation to the dropout are used in the analyses.

* When the shunt is occluded and a vascular anastomosis is re-created surgically.

* When the participant dies or is transferred to another hospital.

* When the participant wishes to terminate his or her participation. (Here, the participant needs to permit data use until the dropout date.)

If some patients drop out, no new patients will be enrolled, and the data will be analysed according to the intention-to-treat principle with use of the FAS cohort.

#### Missing data

Data collection will be interrupted. When the study resumption becomes possible, data collection will be resumed.

* When a participant is hospitalised.

* When the haemodialysis access is narrowed and a percutaneous transluminal angioplasty (PTA) is performed.

* When the haemodialysis access is occluded and blood flow restarts via non-surgical treatment such as massage or PTA.

* When study continuation is difficult because of unavoidable circumstances to participants.

* When study continuation is difficult because of unavoidable circumstances to medical staff.

As a mixed effect model allows for a robust analysis of missing data [70], we will use all available data for the analysis. In the event of any other situations, researchers will decide on a response after consultation.

## Discussion

Repeated pain during haemodialysis access cannulations is a common symptom reported by haemodialysis patients. Music therapy is a safe and inexpensive means for addressing these painful experiences. Music therapy has been effective at alleviating pain and anxiety in a variety of disease contexts [25, 26, 28–30, 33]. Given previous studies, which could be limited by the placebo effect, music therapy could be particularly beneficial to haemodialysis patients [71]. Thus, we propose a crossover trial in order to verify the effectiveness of music therapy for alleviating pain during haemodialysis access cannulations conducted on haemodialysis patients.

The most important characteristic of this trial is the use of a white noise condition as a control comparison. Since several previous studies used a ‘no-sound’ condition as a control but did not address potential placebo effects of the music intervention [37], the effectiveness of previous music therapy interventions could be misleading (Fig. 2). Therefore, the proposed protocol will implement white noise as a way to separate out the potential music effect from any sound-related placebo effect. Although white noise does not have any musical properties, previous studies have observed the relaxation effects of white noise [48, 72]. Furthermore, as stated, we cannot mask the intervention from the patients. However, given that both music and white noise have a relaxation effect, we can inform patients that both music and white noise could help alleviate pain during cannulations. In this way, we will be able to determine whether music mitigates pain above and beyond the implementation of a relaxing sound stimulus. Furthermore, a no-sound period will be implemented during the first and third weeks. As a secondary analysis, white noise and music will be compared with these no-sound conditions.

The planned protocol comprises a crossover randomised controlled trial. VAS pain scores will serve as the primary outcome. Feelings regarding cannulation and the effect of music therapy are likely to differ greatly between individuals. If we were to use a parallel-group trial, there would be concerns that individual differences could mask the treatment effect. Hence, there is quite a bit of variability in past studies concerning the effect of music therapy on cannulation pain. With a crossover randomised controlled trial, it is possible to exclude the influence of individual differences [73–75]; thus, the effect of music therapy can be extracted with a higher power.

We will also evaluate, as secondary outcomes, whether music therapy improves anxiety prior to cannulation and whether BP and HR change at the start of dialysis [29, 30, 33, 76–78]. If music therapy is shown to lower BP in dialysis patients, we might be able to suggest the possibility of using music therapy to treat hypertension in dialysis patients [79, 80]. Furthermore, given that the VAS pain and anxiety scores are a subjective report, objective parameters should be included. Therefore, we will also include S-AMY as a stress marker that can be easily measured to assess patient stress [81].

We will be using Mozart’s Sonata for two pianos in D major (K.448). This piece has been used in various studies assessing the effects of music therapy [46, 82–84]. Some studies suggest that using each patient’s favourite music is more effective than using one type of music [30]. However, if the Mozart piece is effective in reducing pain and anxiety in the proposed study, a single music source could be easily implemented as background music (BGM) within a dialysis room. For practical reasons, each patient’s favourite music could not be used simultaneously as BGM in one setting. Therefore, the observation that one specific musical piece is effective for alleviating pain and anxiety could aid with therapeutic feasibility.

A few study limitations should be noted. First, participants cannot be made blind to their intervention allocation and some of the outcome measures. To address this issue, we will tell participants that both music and white noise are known to facilitate relaxation. Furthermore, all VAS assessments will be conducted without operator assistance. Therefore, all reports will be kept confidential. Second, this is a crossover randomised controlled trial and carry-over effects could occur. To minimise this possibility, we will implement a washout period (1 week) between the white noise and music periods. If carry-over effects emerge, we will use only data from the second week (HD5–7) for the analysis, as it is a randomised parallel-group design. The current sample size was computed on the basis of the single period data alone; thus, the estimated sample size would be sufficient for the period-specific analysis. Third, the proposed study will be somewhat protracted (no fewer than 4 weeks for each patient). Therefore, there is a risk of participant dropout, which could create a selection bias. We will try to alleviate dropout through monetary incentives for participation [85].

The proposed protocol will include a multi-facility, operator-blind, crossover randomised controlled trial to examine whether music therapy successfully reduces cannulation pain for dialysis patients. If predicted results manifest, we should have evidence that patients’ cannulation phobia and pain experiences can be reduced through a safe and cost-effective treatment strategy.

## Trial status

This study received ethical approval from the chief ethics committee at Nagasaki University Hospital on 19 June 2018. This study was registered at University Hospital Medical Information Network Clinical Trials Registry (UMIN-CTR): UMIN 000032850 on 1 July 2018 (https://upload.umin.ac.jp/cgi-bin/ctr/ctr_view_reg.cgi?recptno=R000037447). The first patient was enrolled on 22 August 2018. At the original submission of the protocol, we have completed our pilot study (UMIN000024754). So far, 102 participants have been randomly assigned. Patients are being recruited now and the recruitment was scheduled to be completed at the end of September 2019.

## Supplementary information

**Additional file 1.** Standard Protocol items: Recommendations for Interventional Trials (SPIRIT) 2013 Checklist: recommended items to address in a clinical trial protocol and related documents.

**Additional file 2.** Monte Carlo simulation study for selecting an optimal statistical test.

## Data Availability

The datasets used or analysed (or both) during the proposed protocol will be available from the corresponding author upon reasonable request.

## Abbreviations

BGM: Background music
BP: Blood pressure
FAS: Full analysis set
HD: Haemodialysis
HR: Heart rate
PC: Personal computer
PPS: Per protocol set
PTA: Percutaneous transluminal angioplasty
REDCap: Research electronic data capture
S-AMY: Salivary amylase activity
STAI: State-Trait Anxiety Inventory
VAS: Visual Analogue Scale
