# Supplementary material for: Effectiveness of music therapy for alleviating pain during haemodialysis access cannulation for patients undergoing haemodialysis: a multi-facility, single-blind, randomised controlled trial
Source: Trials. 2019 Nov 19;20:631. doi: 10.1186/s13063-019-3773-x (PMC6862830; doi:10.1186/s13063-019-3773-x)
Supplement: Supplementary file 2 — Additional file 2. Monte Carlo simulation study for selecting an optimal statistical test. [file 13063_2019_3773_MOESM2_ESM.docx]

**Additional File 2.**

**Monte-Carlo simulation study for selecting an optimal statistical test.**

R version 3.3.1 was used for this simulation study. Six VAS measures (3 in the music and other 3 in the white noise period) were generated randomly from a normal distribution with mean of 2.05 and standard deviation of 1.2 based on our pilot study. Variance covariance structure among the 6 measures are generated based on the compound symmetry structure with correlation varied from 0.5, 0.6 and 0.7. Negative values were replaced with zero. Sample size was set to 120 within each sequence. In order to example a magnitude of impact from missing observations, we randomly created missing observation in the following 4 scenarios (1) no missing (2) missing happens only at the 4^th^ measurement, (3) missing happens at both 4^th^ and 5^th^ measurements, and (4) missing happens in all observations in the second period. Percent of missing was set to 20%. For each randomly generated dataset, we applied the following 3 statistical methods.

1) Paired t-test to compare the 2 mean of 3 pain scores between the two periods (a unit of observation in the analysis is the within patient mean score among 3 scores, the analysis includes 2 mean scores per patient).

2) Linear mixed model to compare the 2 means of 3 pain scores between the groups (a unit of observation is the same as 1) )

3) Linear mixed model to compare the 6 pain scores between the two periods. (a unit of observation is the pain scores, the analysis includes 6 observations per patient)

The following graphs and tables show statistical power for each simulation including 1000 randomly generated datasets. Empirical power was computed as the proportion of the datasets among 1000 where statistical significance was observed.

Result of the Monte-Carlo simulation study

Appendix Figure 1. Empirical powers


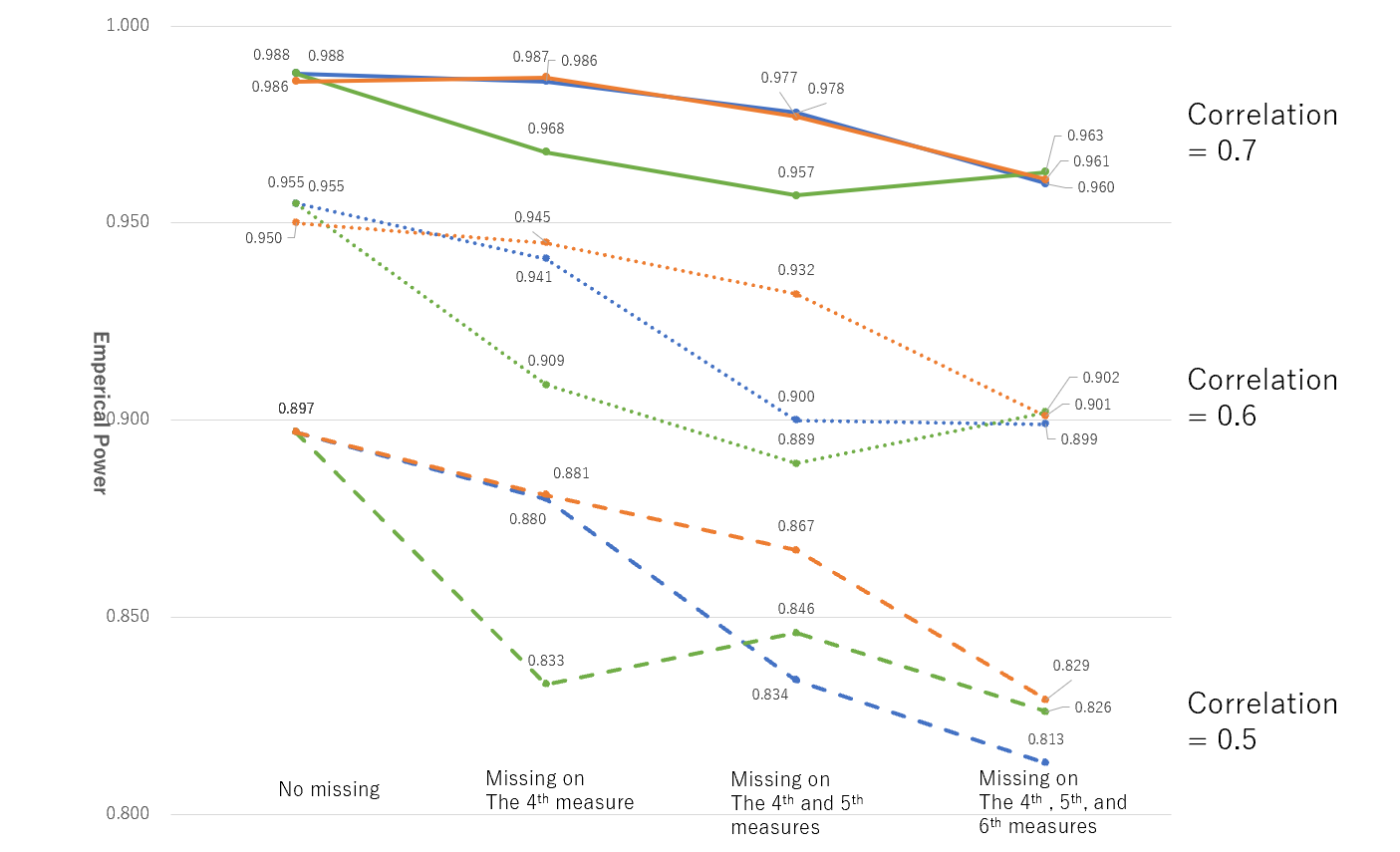


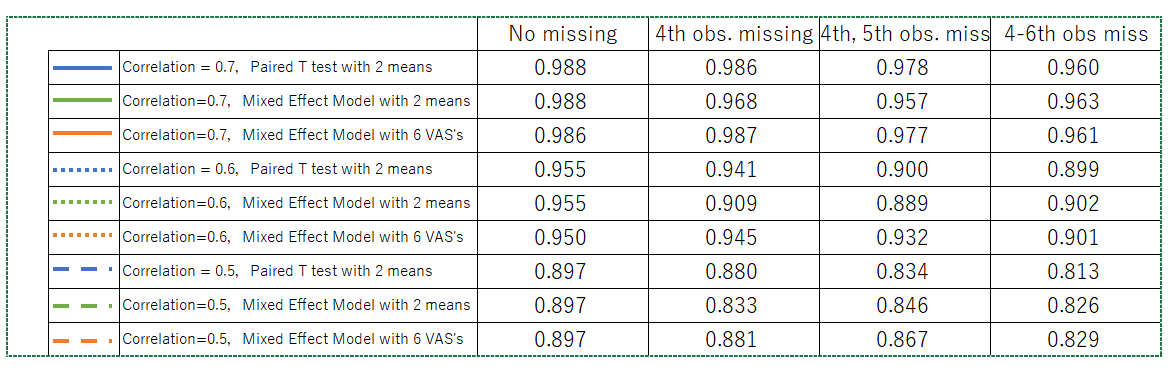
Appendix Table 1. Empirical powers
